# Unique motifs identify PIG-A proteins from glycosyltransferases of the GT4 family

**DOI:** 10.1186/1471-2148-8-168

**Published:** 2008-06-04

**Authors:** Nupur Oswal, Narinder Singh Sahni, Alok Bhattacharya, Sneha Sudha Komath, Rohini Muthuswami

**Affiliations:** 1 School of Life Sciences, Jawaharlal Nehru University, New Delhi – 110 067, India; 2 School of Information Technology, Jawaharlal Nehru University, New Delhi – 110 067, India; 3 Department of Biotechnology, Jaypee Institute of Information Technology, NOIDA, India

## Abstract

**Background:**

The first step of GPI anchor biosynthesis is catalyzed by PIG-A, an enzyme that transfers *N*-acetylglucosamine from UDP-*N*-acetylglucosamine to phosphatidylinositol. This protein is present in all eukaryotic organisms ranging from protozoa to higher mammals, as part of a larger complex of five to six 'accessory' proteins whose individual roles in the glycosyltransferase reaction are as yet unclear. The PIG-A gene has been shown to be an essential gene in various eukaryotes. In humans, mutations in the protein have been associated with paroxysomal noctural hemoglobuinuria. The corresponding PIG-A gene has also been recently identified in the genome of many archaeabacteria although genes of the accessory proteins have not been discovered in them. The present study explores the evolution of PIG-A and the phylogenetic relationship between this protein and other glycosyltransferases.

**Results:**

In this paper we show that out of the twelve conserved motifs identified by us eleven are exclusively present in PIG-A and, therefore, can be used as markers to identify PIG-A from newly sequenced genomes. Three of these motifs are absent in the primitive eukaryote, *G. lamblia*. Sequence analyses show that seven of these conserved motifs are present in prokaryote and archaeal counterparts in rudimentary forms and can be used to differentiate PIG-A proteins from glycosyltransferases. Using partial least square regression analysis and data involving presence or absence of motifs in a range of PIG-A and glycosyltransferases we show that (i) PIG-A may have evolved from prokaryotic glycosyltransferases and lipopolysaccharide synthases, members of the GT4 family of glycosyltransferases and (ii) it is possible to uniquely classify PIG-A proteins versus glycosyltransferases.

**Conclusion:**

Besides identifying unique motifs and showing that PIG-A protein from *G. lamblia *and some putative PIG-A proteins from archaebacteria are evolutionarily closer to glycosyltransferases, these studies provide a new method for identification and classification of PIG-A proteins.

## Background

Biosynthesis of glycosylphosphatidylinositol (GPI) anchor in the endoplasmic reticulum (ER) of the cell represents a highly conserved activity in eukaryotes due to the conservation of the basic structural unit of GPI anchors [1,2]. The basic anchor consists of a phosphatidylinositol (PI) moiety decorated with a glucosamine (GlcN) to which additional 3–5 mannose (Man) residues are attached to generate a linear chain. One or more of these Man residues are in turn modified by ethanolamine phosphate (EtP). The nascent protein destined to be GPI anchored is attached to the EtP present on the third Man [3].

Despite the overall structure conservation, several species-specific differences exist within the GPI biosynthetic pathway. The number of Man residues varies from species to species. For example, GPI anchors isolated from *T. cruzi *and *P. falciparum *possess an additional mannose residue [4]. The EtP modification of the Man residues also shows significant species-dependent variation [5]. GPI anchors from many species contain additional sugars such as galactose (Gal) attached to some of the Man residues. In addition, branching at the sugar residues has also been reported [6]. The inositol too could have additional acylation at the 2'-OH position in some species and lipid remodeling of the GPI anchors can add to the possible variations observed in the glycolipid anchors of several species [7].

The advantages of anchoring proteins via GPI anchors vary depending on the protein anchored and the organism concerned [8]. Unlike integral membrane proteins, GPI anchored proteins can be readily released from the cell surface under appropriate conditions. In *C. neoformans*, for instance, GPI anchor has been postulated to regulate the secretion of phospholipase B1 in response to environmental conditions and hence determine virulence [9]. The shedding of several GPI anchored proteins from the sperm cell surface by the GPIase activity of angiotensin converting enzyme has been shown to be crucial for fertilization in mice [10]. Anchoring of proteins to the membrane via a glycolipid anchor also allows for greater three-dimensional flexibility for the protein on the cell surface and can influence rates of ligand-interaction [11]. Thus, several GPI-anchored proteins act as cell-surface receptors. For example, the LPS receptor in human endothelial membrane is GPI anchored and its removal with PI-specific phospholipase C (PI-PLC) affects leukocyte recruitment [12]. Similarly, the malarial parasite receptor on erythrocytes also is GPI anchored [13].

As cell surface receptors, GPI anchored proteins play a vital role in cell signaling, growth, adhesion, and virulence. Lowering the expression of such proteins or interference with GPI anchor synthesis would, therefore, be expected to interfere with several important functions of the cell. Indeed, tethering of cell surface proteins using GPI anchors appears to be critical for the normal development and functioning of eukaryotes including many disease-causing organisms (for a recent review see [14]).

GPI anchor biosynthesis is a multi-step process involving at least 23 proteins in humans. The first step of this pathway involves transfer of N-acetylglucosamine (GlcNAc) from UDP-GlcNAc to PI, a reaction catalyzed by PIG-A. The gene coding for PIG-A has been cloned from many organisms and has been demonstrated to be essential for cell viability [15-18]. Using bioinformatics tools, we have attempted to understand the evolution of PIG-A. Our results suggest that it has evolved from glycosyltransferases present in prokaryotic systems. We have also identified motifs unique to PIG-A that may be helpful to characterize PIG-A proteins from newly sequenced genomes.

## Results

### GPI-GnT complex: Species-dependent variation

The first step in the GPI anchor biosynthesis involves the GlcNAc transferase complex (GPI-GnT). As mentioned before this complex comprises of seven proteins in humans. PIG-A has been hypothesized as the catalytic unit of the GPI-GnT complex due to the presence of a conserved glycosyltransferase domain. Using the human sequences as query, BLAST analysis was carried out to identify homologous sequences in other eukaryotes (see table 1 for the list of organisms and proteins surveyed along with the abbreviations used). The results show the presence of PIG-A in all organisms [19,20].

**Table 1:** List of organisms and proteins surveyed.

*Homo sapiens*	HS	PIG-A	HSPA	Eukarya
*Rattus norvegicus*	RN	PIG-A	RNPA	Eukarya
*Entamoeba histolytica*	EH	PIG-A	EHPA	Eukarya
*Candida albicans*	CA	PIG-A	CAPA	Eukarya
*Saccharomyces cerevisiae*	SC	PIG-A	SCPA	Eukarya
*Schizosaccharomyces pombe*	SP	PIG-A	SPPA	Eukarya
*Leishmania major*	LM	PIG-A	LMPA	Eukarya
*Giardia lamblia*	GL	PIG-A	GLPA	Eukarya
*Drosophila melanogaster*	DM	PIG-A	DMPA	Eukarya
*Plasmodium falciparum*	PF	PIG-A	PFPA	Eukarya
*Dictyostelium discoideum*	DD	PIG-A	DDPA	Eukarya
*Trypanosoma brucei*	TB	PIG-A	TBPA	Eukarya
*Oryza sativa*	OS	PIG-A	OSPA	Eukarya
*Arabdopsis thaliana*	AT	PIG-A	ATPA	Eukarya
*Caenorhabditis elegans*	CE	PIG-A	CEPA	Eukarya
*Paramecium tetraurelia*	PT	PIG-A	PTPA	Eukarya
*Aeropyrum pernix*	AP	PIG-A	APPA	Archaea
*Thermoplasma acidophilum*	TA	PIG-A	TAPA	Archaea
*Methanosarcina barkeri*	MB	PIG-A	MBPA	Archaea
*Methanosarcina acetivorans*	MT	PIG-A	MTPA	Archaea
*Bacteroides thetaiotaomicron*	BT	Glycosyltransferase	BTGT	Bacteria
*Clostridium beijerincki*	CB	Glycosyltransferase	CBGT	Bacteria
*Alkaliphilus metalliredigenes*	AM	Glycosyltransferase	AMGT	Bacteria
*Mannheimia succiniciproducens*	MS	Glycosyltransferase	MSGT	Bacteria
*Actinobacillus succinogenes*	AS	Glycosyltransferase	ASGT	Bacteria
*Propionibacterium acnes*	PA	Glycosyltransferase	PAGT	Bacteria
*Pyrococcus furiosus*	PF	Glycosyltransferase	PFGT	Archaea
*Desulfitobacterium hafniense*	DH	Glycosyltransferase	DHGT	Bacteria
*Clostridium tetani*	CT	Glycosyltransferase	CTGT	Bacteria
*Methanosarcina acetivorans*	MA	Glycosyltransferase	MAGT	Archaea
*Mycobacterium sp*.	MY	Glycosyltransferase	MYGT	Bacteria
*Cryptosporidium parvum*	CP	Glycosyltransferase	CPGT	Eukaryote
*Methanothermobacter thermautotrophicus*	MT	LPS glycosyltransferase	MTLT	Archaea
*Bacteroides thetaiotaomicron*	BT	LPS glycosyltransferase	BTLT	Bacteria
*Bacillus halodurans*	BH	Glycosyltransferase	BHGT	Bacteria

The early-branching eukaryote *G. lamblia *possesses a very rudimentary endoplasmic reticulum [21]. Since most of the GPI-anchor biosynthetic enzymes are localized in endoplasmic reticulum an analysis was carried out to find out which of the polypeptides of the GPI-GnT complex are present in *G. lamblia*. Our analysis revealed presence of only PIG-A but not any of the accessory proteins in this organism (Table 2). Thus, it appears that PIG-A is sufficient for the formation of GlcNAc-PI. Therefore, we decided to investigate the evolution of PIG-A with the aim of understanding its ancestral sequences.

**Table 2:** Proteins present in GPI-GnT complex.

	PIG-A	PIG-P	PIG-C	PIG-Q	PIG-H	DPM2	PIG-Y
HS	Yes	Yes	Yes	Yes	Yes	Yes	Yes
RN	Yes	Yes	Yes	Yes	Yes	Yes	Yes
EH	Yes	Yes	Yes	Yes	No	No	No
CA	Yes	Yes	Yes	Yes	Yes	No	Yes (Eri1)
SC	Yes	Yes	Yes	Yes	Yes	No	Yes (Eri1)
SP	Yes	Yes	Yes	Yes	Yes	Yes	Yes (Eri1)
LM	Yes	Yes	Yes	Yes	Yes	Yes	No
GL	Yes	No	No	No	No	No	No
DM	Yes	Yes	Yes	Yes	Yes	No	No
PF	Yes	Yes	Yes	Yes	Yes	No	No
DD	Yes	Yes	Yes	Yes	Yes	Yes	No
TB	Yes	Yes	Yes	Yes	Yes	Yes	No
OS	Yes	Yes	Yes	Yes	Yes	Yes	No
AT	Yes	Yes	Yes	Yes	Yes	Yes	No
CE	Yes	Yes	Yes	Yes	No	No	No
PT	Yes	ND	ND	ND	ND	ND	ND

BLAST analysis identified only PIG-A from *P. tetraurelia *as this has gene has been cloned and studied. The genome of *P. tetraurelia *has not been sequenced and annotated. Therefore, we have not been able to study the presence of PIG-H, PIG-C, PIG-P, PIG-Q, PIG-Y, and DPM2 in this organism.

**Figure 1:**
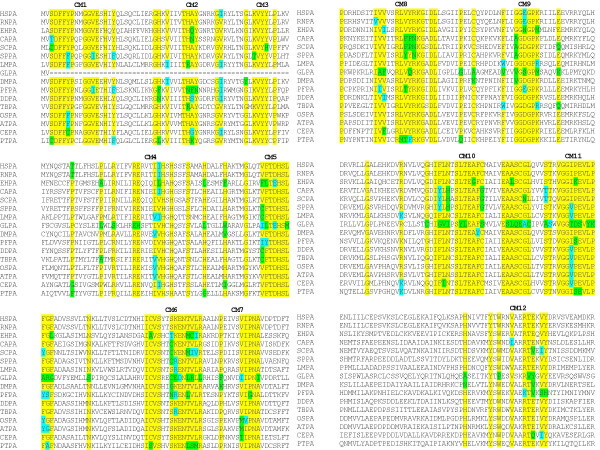
**Identification of conserved motifs in PIG-A protein from eukaryotes**. Clustal W analysis using MAFFT identified twelve conserved motifs in PIG-A protein. Three of these motifs are absent in *G. lamblia*.

**Figure 2:**
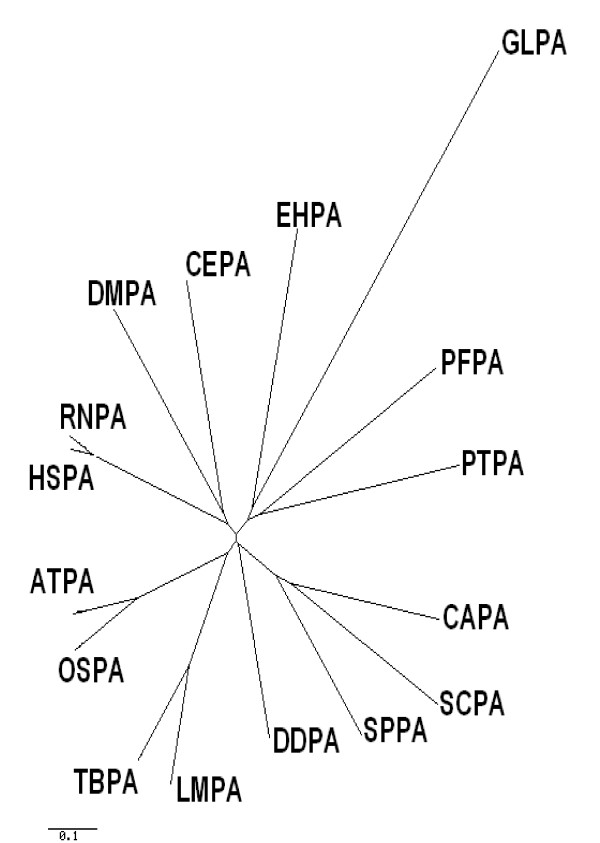
**Phylogenetic analysis of PIG-A protein from eukaryotes**. Phylogenetic tree was constructed using phylodendron program. *G. lamblia *appears to have diverged away from other eukaroyotic PIG-A proteins.

After aligning the sequences using ClustalW (Figure 1), we made an initial inference on the evolution of PIG-A by phylogenetic analysis (Figure 2); [see Additional file 1]. The *G. lamblia *sequence was found to be relatively closer to other protozoan sequences. The fungal (*S. cerevisiae*, *S. pombe*, and *C. albicans*) PIG-A proteins clustered together. Similarly, the PIG-A proteins from *L. major *and *T. brucei *appeared to have diverged from a common ancestor. Surprisingly *P. falciparum *and *E. histolytica *were grouped together and far from the kinetoplastid PIG-As in spite of their different phylogenetic relationship. PIG-As from all higher eukaryotes were found in the same cluster, with plants forming a distinct subgroup within this cluster with the exception of the protozoan *D. discoideum*, which was observed to be closer to the higher rather than the lower eukaryotes.

**Figure 3:**
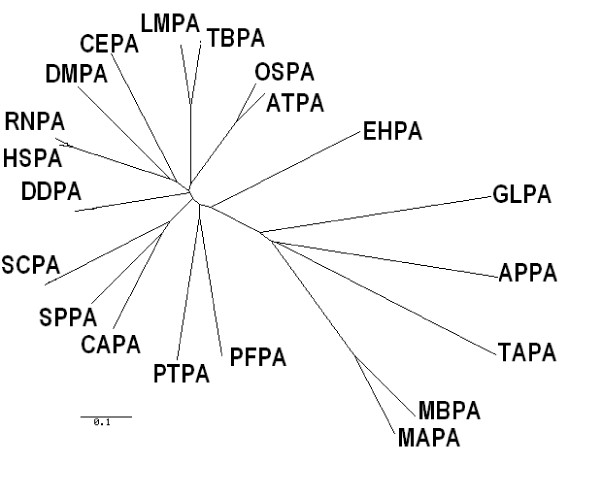
**Phylogenetic analysis of PIG-A protein from archaeabacteria and eukaryotes**. Phylogenetic tree was constructed using phylodendron program. The giardial protein appears to be closer to the archaeal proteins than to other eukaryotic PIG-A proteins.

Since the presence of GPI-anchored proteins in some species of archaea has been reported earlier [22,23], sequences from the archaeal genome database that showed significant similarity with the consensus PIG-A sequence from eukaryotes were identified by BLAST. A phylogenetic analysis including putative PIG-As from some archaeal species such as *A. pernix*, *T. acidophilum, M. barkeri *and *M. acetivorans *along with the eukaryotic PIG-A sequences showed that the *G. lamblia *PIG-A was closer to its archaeal counterparts rather than other eukaryotes, including many protozoa (Figure 3); [see Additional file 2].

### Motif identification in PIG-A

A discovery approach was used to identify motifs in PIG-A sequences that could potentially be used to identify its ancestors. In order to identify conserved residues and potential motifs a number of these sequences were aligned and the alignment was pruned manually by removing gaps.

Motifs were designated as stretches of amino acid residues where some of the amino acid residues were conserved and represented in the same format as that in "Prosite database" [24,25]. These were labeled as conserved motifs (CM1-12) and numbered from N- to C-terminal of the protein as in Prosite format. Twelve motifs were identified by this method. The motifs were subsequently verified by using PRATT [26,27] and Scan Prosite as explained in "Methods". Some of the motifs were also modified and the modified versions of the motifs were labeled with an additional small alphabet. For example, the modified version of CM1 was labeled as CM1a. Modifications were done based on sequence conservation in some of the eukaryotic PIG-A sequences. The list of conserved motifs, identified in this study by aligning eukaryotic PIG-A sequences, is given in Table 3. In addition, Gblocks [28,29] was also employed for the identification of the motifs and the results agreed well with the manual approach [see Additional file 3]. Only small differences were discernable, for example, CM7 was identified by manual method as **[PFK]**-X-X-X-X-[VIMT]-[VI]-[PG]-N-[AI] and by Gblocks software as [VISCL]-[LVMI]-R-[ATSGH]-X-X-X-**[PKQ]**-X-X-[VIA]-[SFYD]-[VIMT]-[VI]-[PG]-N-[AI]-[VLTI].

**Table 3:** Conserved motifs in PIG-A proteins from eukaryotes.

MOTIF #	MOTIF SEQUENCE
CM1	[STC]-D-F-F-[YFC]-P-X-X-G-G-[VI]-E-X-H-X-[YF]
CM2	G-[HFNL]-[KRS]-[VI]-[VI]-[ITV]-X-T-[HRN]-[AQNFSGK]-[YN]-X-X-[RTC]-X-G-[VI]
CM3	[GY]-[LIM]-[KT]-V-Y-[YH]-X-P
CM4	[PLA]-X-X-[RS]-X-[ILV]-[FLVH]-[VIRLY]-[RE]-[EH]-X-[IVF]-X-[IV]-[ILV]-H-[SGAC]-H-[GQSA]-[STAN]-[FLATY]-S
CM5	G-X-[KPQRS]-[TAV]-[VFCI]-[FLY]-T-[DE]-H-S-[LM]
CM6	I-[CAS]-V-S-X-[TCEIV]-[STCGN]-[KRE]-[ED]-N-[TML]-[VCIRS]-[LVIM]-[RL]
CM7	[PFK]-X-X-X-X-[VIMT]-[VI]-[PG]-N-[AI]
CM8	[IV]-[VAI]-[VIF]-[VILMA]-X-R-[LM]-[VYFT]-[YPQF]-[RN]-K-G-X-D-L
CM9	[FWVY]-[ILVY]-[VI]-[GAV]-G-[EDNS]-G-P-[KMR]
CM10	[GC]-[HDQ]-I-[FYG]-[LIV]-[NHI]-X-S-[LY]-[TL]-E-[AG]-[FY]-[CGS]-X-[AVIS]-[IL]-[VIL]-E-[AS]-[AL]-[SQ]-[CE]-[GNA]-[LC]
CM11	[STA]-[TS]-X-V-G-G-[IVT]-[PDSK]-[ES]-V-[LY]-[PK]
CM12	Y-[STDN]-[WP]-X-X-[VI]-[AS]-X-[RK]-[TV]-[EVYQ]-X-[VIS]-[YH]

### Distribution of the motifs in eukaryotic PIG-A

In general, the twelve motifs identified by us were found in all eukaryotic PIG-As except *G. lambia*. Three of the motifs (CM1, CM2, and CM3) were not detected in *G. lamblia *(Table 3). Since *G. lamblia *PIG-A is smaller than other PIG-A proteins and lacks the three N-terminal motifs it is likely that these motifs were added to the eukaryotic PIG-A after *G. lamblia *evolved. The other possibility is that there has been a deletion in the *G. lamblia *gene during the course of evolution. This possibility cannot be ruled out as these motifs were found in archaeal PIG-A (see subsequent sections).

### Archaeal PIG-A and distribution of motifs

**Figure 4:**
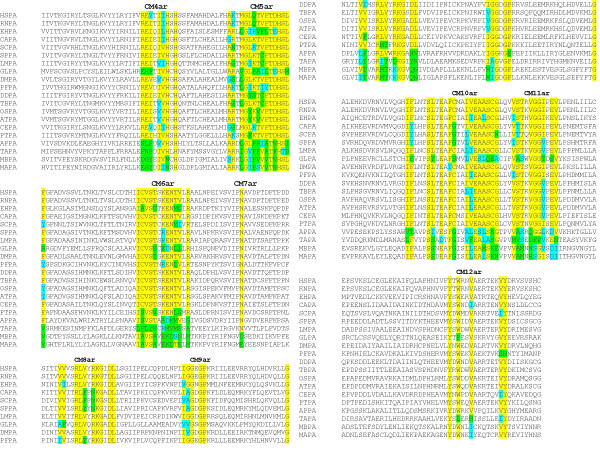
**Identification of conserved motifs in PIG-A proteins from archaeabacteria and eukaryotes**. Clustal W analysis of PIG-A protein from archaeabacteria and eukaryotes using MAFFT led to the identification of conserved motifs in these proteins.

Having identified sequences from the archaeal genome database that showed significant similarity with the consensus PIG-A sequence from eukaryotes, motifs were also deciphered using an alignment of eukaryotic and putative PIG-A sequences from some archaeal species as described before (Figure 4). Motifs related to CM4, 5, 6, 8, 9, 10, 11 and 12 described before for eukaryotic PIG-A were identified using both manual as well as Gblocks software (Figure 4); [see Additional file 4]. The motifs identified manually by aligning eukaryotic and archaeal PIG-A sequences were labeled with a suffix 'ar' (Table 4). For example, CM3 and CM3ar are the conserved motifs identified by aligning all the eukaryotic PIG-A sequences and both the eukaryotic and archaeal PIG-A sequence respectively. As shown in Table 4, there were discernable differences in these motifs as compared to those identified by alignment of eukaryotic PIG-As alone (compare Table 3 and Table 4). However, we could not discern any pattern in the amino acid substitutions leading to alteration in motifs.

**Table 4:** Conserved motifs in PIG-A proteins after aligning the eukarya and archaea sequences.

MOTIF #	MOTIF SEQUENCE
CM1ar	D-[FTW]-[FHY]-[YFCP]-[PS]-X-X-[GD]-G-[VI]
CM2ar	G-[HNLFY]-X-[VI]-[VISMH]-[ITV]-[VIMF]-[TS]-[HRVN]-X-[YNLG]
CM3ar	[VIK]-[YVI]-X-X-[PK]
CM4ar	[RESFD]-[EHLNG]-[VIPYF]-X-[IV]-[IV]-[HN]-X-H
CM5ar	[AGS]-[KRNGS]-X-[MVLI]-G-X-[KPQRS]-X-X-X-T-[DENF]-H-[ST]-[LMID]-[FAYV]
CM6ar	[IL]-[CASF]-[VL]-[SY]-X-X-[KREA]-[EDMK]-[NKVD]-[TMLS]-X-X-[RGAM]
CM7ar	[NDE]
CM8ar	[VAIL]-X-X-X-R-[LMI]-[VYFT]-X-[RNDK]-K-G-X-[DHYQ]-[LVNR]
CM9ar	[IVM]-[GAIV]-G-X-G-[PE]
CM10ar	[IVL]-[FYGT]-X-X-X-S-[LYIS]-X-[ED]-[ASGT]-[FY]-[CGS]-X-X-[ILAV]-[VILF]-E-[AS]-[ALMI]-[SQA]-[CESK]-[GNAE]
CM11ar	[VIM]-[STAV]-[TSM]-X-[VQDNH]-[GFS]-[GP]-[IVTL]-X-[EDS]-[VNI]
CM12ar	Y-X-[WPL]-X-X-[VIH]-X-X-X-X-X-X-[VIS]-[YH]

### PIG-A and Glycosyltransferases

Glycosyltransferases have been classified into 90 groups based on amino acid sequence similarity by Coutinho *et al*. [30] and are listed in the CAZy web site [31]. This method of classification also reflects the molecular mechanism of catalysis within a given family. In such a classification, PIG-A belongs to the GT4 family of glycosyltransferases comprising of, amongst other members, liposaccharide biosynthesis RfbU-related protein and polysaccharide biosynthesis protein (for example, NP_616007 from *Methanosarcina acetivorans*) involved in cell wall biogenesis. All PIG-A proteins possess a conserved glycosyltransferase domain with the conserved EX_7_E motif.

**Figure 5:**
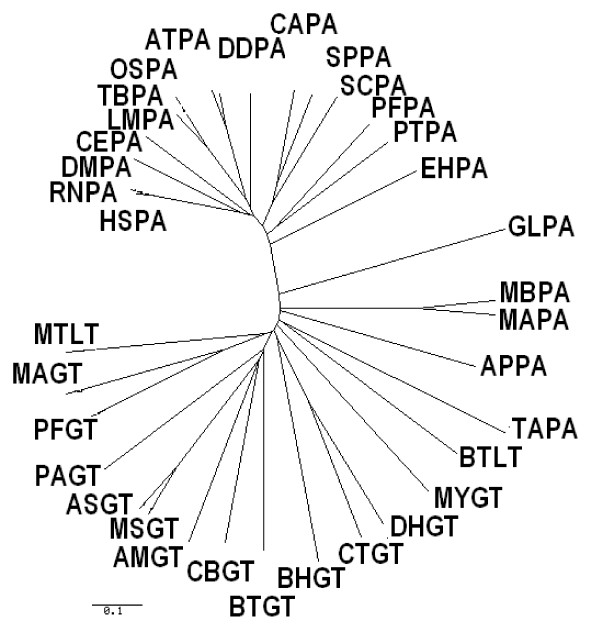
**Phylogenetic analysis of PIG-A and glycosyltransferase proteins from prokaryotes, archaeabacteria, and eukaryotes**. Phylogenetic analysis using phylodendron program confirms that the PIG-A protein from eukaryotes are evolutionarily a separate branch of proteins.

**Figure 6:**
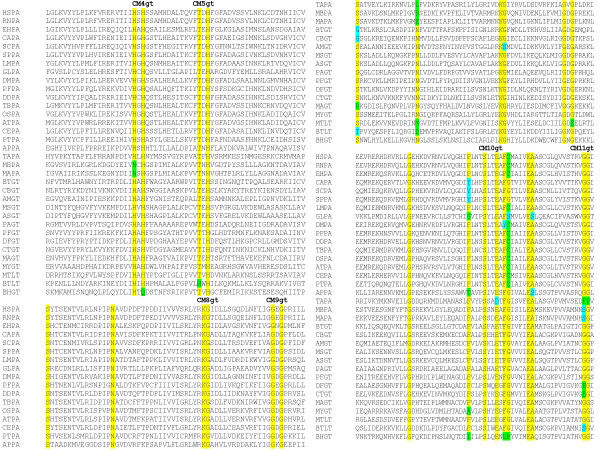
**Identification of conserved motifs in PIG-A and glycosyltransferases proteins from prokarotes, archaeabacteria, and eukaryotes**. Clustal W analysis using MAFFT was done to identify conserved motifs in PIG-A and glycosyltransferase proteins from prokaryotes, archaeabacteria, and eukaryotes.

Archaeal and bacterial glycosyltransferases belonging to GT4 family were used for multiple sequence alignment with PIG-A sequences to understand the phylogenetic relationship within the family (Figure 5); [see Additional file 5]. This alignment showed the presence of six conserved motifs (Figure 6; Table 5). The motifs identified manually were labeled with a suffix 'gt' to denote that these motifs were identified by alignment of PIG-A sequences with glycosyltransferases. The numbering corresponds to the motifs whose progenitors they appeared to be. Thus, the motif CM4gt is the progenitor of CM4 (compare Table 3 and Table 5). The motif CM10gt, [FYGTAL]-X-X-X-S-X-X-[ED]-X-[FLY]-[CSGP]-X-X-X-X-E-[AS], is a specific form of EXFXXXXXE motif present in many glycosyltransferases, including α-mannosyltransferases, where the consensus sequence for this motif has been identified as SXXEFGLPXXE [32]. Motifs CM1, 2, 3, 6, 7, and 12 or their variants were not detected. Interestingly, in this respect, *G. lamblia *PIG-A appeared to be similar to glycosyltransferases and LPS synthesizing enzymes.

**Table 5:** Conserved motifs in PIG-A proteins after aligning the eukarya and archaea PIG-A sequences with bacterial and archeal glycosyltransferases.

MOTIF #	MOTIF SEQUENCE
CM4gt	[HN]-X-[HQ]
CM5gt	[TH]-X-H
CM8gt	K-[GS]
CM9gt	G-X-[GE]
CM10gt	[FYGTAL]-X-X-X-S-X-X-[ED]-X-[FLY]-[CSGP]-X-X-X-X-E-[AS]
CM11gt	[GFSES]-[GP]

### Motif analysis using ScanProsite

The conserved motifs identified by aligning eukaryotic PIG-A were further analyzed using ScanProsite tool to determine whether these motifs are characteristics of PIG-A or whether they are present ubiquitously in glycosyltransferases and possibly other proteins. All the motif sequences and the alterations done to the consensus motif sequences (as discussed in the section on motif discovery) are listed in Table 6.

**Table 6:** Sequences of motifs used for PLSR analysis.

Motif	Sequence
CM1	[STC]-D-F-F-[YFC]-P-X-X-G-G-[VI]-E-X-H-X-[YF]
CM1a	D-[FTW]-[FHY]-[YFCP]-[PS]-X-X-[GD]-G-[VI]
CM1b	[STC]-D-F-F-[YFC]-P-X-X-G-G-[VI]
CM1c	G-G-[VI]-E-X-H-X-[YF]
CM1d	D-[FTW]-[FHY]-[YFCP]-[PS]-X-X-[GD]-G-[VI]-[EQS]-X-[HYS]
CM2	G-[HFNL]-[KRS]-[VI]-[VI]-[ITV]-X-T-[HRN]-[AQNFSGK]-Y-X-X-[RTC]-X-G-[VI]
CM2a	G-[HNLFY]-X-[VI]-[VISMH]-[ITV]-[VIMF]-[TS]-[HRVN]-X-Y
CM2b	G-[HNLFY]-X-[VI]-[VISMH]-[ITV]-[VIMF]-[TS]-[HRVN]-X-[YNLG]
CM2c	G-[HFNLY]-[KRS]-[VI]-[VISMH]-[ITV]-[VIMF]-[TS]-[HRVN]-[AQNFSGK]-[YNLG]
CM2d	G-[HFNL]-[KRS]-[VI]-[VI]-[ITV]-X-T-[HRN]-[AQNFSGK]-Y
CM3	[GY]-[LIM]-[KT]-V-Y-[YH]-X-P
CM4	[PLA]-X-X-[RS]-X-[ILV]-[FLVH]-[VIRLY]-[RE]-[EH]-X-[IVF]-X-[IV]-[ILV]-[GAC]
CM4b	[RE] – [EH] – [RQNSKE] – [VIF] – X – [IV] – [ILV] – H – [SAGC] – H
CM4c	[RE]-[EH]-[RQNSKE]-[VIF]-X-[IV]-[ILV]-H-[SAGC]-H-X-X-X-S
CM4d	[FLVH]-X-[RE]-[EH]-[RQNSKE]-[VIF]-X-[IV]-[ILV]-H-[SAGC]-H-X-X-X-S
CM4e	[ILV]-[FLVH]-X-[RE]-[EH]-[RQNSKE]-[VIF]-X-[IV]-[ILV]-H-[SAGC]-H-X-X-X-S
CM5	G-X-[KPQRS]-[TAV]-[VFCI]-[FLY]-T-[DE]-H-S-[LM]
CM5a	G-X-[KPQRS]-[TAV]-[VFCI]-[FLY]-T-[DE]-H-S-[LM]-[FYA]-[GRS]-[FLG]
CM5b	G-[LIYFV]-[QRKPS]-X-X-[FLYA]-T-[DENF]-H-[ST]-[LMID]
CM5c	G-[LIYFV]-[QRPSK]-[TVARPS]-[VFCI]-[FLYAV]-T-[DENF]-H-[ST]-[LIMD]
CM5d	G-X-[QRPSK]-[TVARPS]-[VFCI]-[FLYAV]-T-[DENF]-H-[ST]-[LIMD]
CM5e	G-X-[QRPSK]-X-[VFCI]-[FLYAV]-T-[DENF]-H-[ST]-[LIMD]
CM6	I-[CAS]-V-S-X-[TCEIV]-[STCGN]-[KRE]-[ED]-N-[TML]-[VCIRS]-[LVIM]-[RL]
CM6e	[TCEIV]-[STCGN]-[KRE]-[ED]-N-[TML]-[VCIRS]-[LVIM]-[RL]
CM6f	V-S-X-[TCEIV]-[STCGN]-[KRE]-[ED]-N-[TML]-[VCIRS]-[LVIM]-[RL]
CM6g	[CAFS]-V-S-X-[TCEIV]-[STCGN]-[KRE]-[ED]-N-[TML]-[VCIRS]
CM8	[IV]-[VAI]-[VIF]-[VILMA]-X-R-[LM]-[VYFT]-[YPQF]-[RN]-K-G-X-D-L
CM8a	[IV]-[VAI]-[VIF]-X-X-R-[LM]-X-[YPQF]-[RN]-K-G-X-D-L
CM8b	[IV]-[VAI]-[VIF]-X-X-R-[LM]-X-X-[RN]-K-G-X-D-L
CM8e	[VILMA]-X-R-[LM]-[VYFT]-[YPQF]-[RN]-K-G-X-D-L
CM8f	X-X-R-[LM]-X-[YPQF]-[RN]-K-G-X-D-L
CM8g	R-[LM]-X-[YPQF]-[RN]-K-G-X-D-L
CM9	[FWVY]-[ILVY]-[VI]-[GAV]-G-[EDNS]-G-P-[KMR]
CM10	[GC]-[HDQ]-I-[FYG]-[LV]-[NHI]-X-S-[LY]-T-E-[AG]-[FY]-[CGS]-X-[AVIS]-[IL]-[VI]-E-[AS]-[AL]-[SQ]-[CE]-[GNA]-[LC]
CM10a	S-[LY]-T-E-[AG]-[FY]-[CGS]-X-[AVIS]-[IL]-[VI]-E-[AS]-[AL]-[SQ]-[CE]-[GNA]-[LC]
CM10b	[GC]-[HDQ]-I-X-[LV]-[NHI]-X-S-[LY]-T-E-[AG]-[FY]-[CGS]-X-X-[IL]-[VI]-E-[AS]-[AL]-[SQ]-[CE]-[GNA]-[LC]
CM10c	[GCA]-X-[IVL]-[FYGT]-[LVIA]-X-X-S-[LYS]-[TLAND]-E-[AGST]-[FY]-[CGS]-X-X-[ILVA]-[VIFL]-E-[AS]
CM10d	[GCA]-X-[IVL]-[FYGT]-[LVIA]-X-X-S-[LYS]-X-E-[AGST]-[FY]-[CGS]-X-X-[ILVA]-[VIFL]-E-[AS]-[ALMI]-[SQA]
CM11	[STA]-[TS]-X-V-G-G-[IVT]-[PDSK]-[ES]-V-[LY]-[PK]
CM11a	[STA]-[TS]-X-V-G-G-[IVT]-X-[ES]-V-[LY]-[PK]
CM11b	V-G-G-[IVT]-X-[ES]-V-[LY]-[PK]
CM12	Y-[STDN]-[WP]-X-X-[VI]-[AS]-X-[RK]-[TV]-[EVYQ]-X-[VIS]-[YH]
CM12c	Y-X-[WP]-X-X-[VI]-[AS]-X-[RK]-[TV]-X-X-[VIS]-[YH]

CM7 was found to be a promiscuous sequence present in more than 1000 proteins. Therefore, CM7 cannot be used for identification of PIG-A sequences. As pointed out before CM1, CM2, and CM3 were present only in eukaryotic PIG-A sequences except *G. lamblia*. However CM1a and CM2a, modified versions of CM1 and CM2, were also found to be present in archaeal PIG-A sequences (Table 6). It should be noted that CM1a and CM2a are identical to CM1ar and CM2ar, thus confirming that archaeal sequences contain a version of CM1 and CM2. CM2a was also found to be present in many glycosyltransferases, histidine kinases, and transcription regulator Lac I family in addition to PIG-A sequences. All alterations to CM3 resulted in identification of more than 1000 protein suggesting that any modification to CM3 results in a promiscuous sequence that cannot be used for the identification of PIG-A.

As the CM4 motif was long, we split it into two segments for analysis. CM4a as well as CM4e identified only PIG-A sequences including that from *G. lamblia*. These motifs were not present in any other protein sequences including bacterial glycosyltransferases as well as archaeal PIG-A sequences.

CM6, CM6a, and CM6b were found in all eukaryotic PIG-A including *G. lamblia*. Similarly CM8 and CM9 and their modifications were found in all eukaryotic PIG-A including *G. lamblia*. All these motifs were absent in bacterial and archaeal proteins. CM5b, CM5c, CM5d, CM10b, CM10c, and CM12c motifs were also present in glycosyltransferases. CM11b was found in glycogen synthase, a member of the GT3 family, in addition to PIG-A sequences.

This data was used to generate a matrix for analysis by partial least square regression analysis [see Additional file 6].

### Modelling and variable selection

Presence and absence of different motifs in the glycosyltransferases and PIG-A proteins of a large number of prokaryotic and eukaryotic proteins was analysed using a statistical method, partial least squares regression (PLSR). PLSR is particularly well suited to multivariate data analysis, and has lately been used for analysis of genome wide expression data [33] (for a recent review see [34]). A detailed description is given in the section on "Methods".

For the sake of this analysis, each variant of a motif is used as an independent variable. Thus a total of 43 variables were used in the PLSR analysis. A list of this is given in Table 6. An attempt was made to classify the proteins on the basis of a binary label, that is presence or absence of a motif in a set of PIG-A and glycosyl transferases from a number of different species [see Additional file 6 for the matrix obtained as well as the list of genes and their accession numbers]. PLSR was used to understand the association of motifs with different protein lineages. A two-level cross-validation scheme called double cross-validation (DCV) was employed to obtain honest error results. Table 7 shows the confusion matrix for the results obtained on the basis of the 10-fold double cross validation (DCV) approach. The result is based on DCV segments with a total error rate of 23%. The list of motifs that were found useful for classification, that is, the most significant in the first six DCV segments is shown in Table 8. As such, all the variables (motifs) in each DCV segment should be considered to be equally important. The results here correlate well with the results obtained from motif analysis using Scan Prosite. Thus CM1, CM1a and CM1b are important variables in all 10 DCVs, suggesting that these 3 motifs are the most robust of all and are present in all archaeal as well as eukaryotic PIG-A proteins; likewise, CM2c appears as the important variable in 9 out of 10 DCVs and so on. Thus, it is possible to use these 10 motifs (or variables) to classify PIG-A proteins and differentiate them from glycosyltransferases. According to this analysis, all the glycosyltransferases have been correctly classified. Most of the PIG-A proteins have been correctly classified except for a set of twelve proteins. These include PIG-A proteins from *D. rerio, E. cuniculli, M. acetivorans, T. volcanium, P. abyssi, M. barkeri, A. pernix, T. parva, T. gondii, C. neoformans, P. chabaudi and G. lamblia*. In other words, these putative PIG-A proteins should be more appropriately classified as glycosyltransferases. These results agree well with the phylogenetic analysis where the PIG-A protein from *G. lamblia *appears to be more closely related to those from archaeal rather than to those from higher eukaryotes.

**Table 7:** The table shows the confusion matrix for the results obtained using PLSR.

			Known Class	
		PIG-A		GT4
	PIG-A	31/43		12/43
Predicted Class				
	GT4	-		10/10

**Table 8:** The thirteen variables selected as significant in the first six DCV segments.

1.	CM1, CM1a, CM1b
2.	CM2c
3.	CM4e, CM5e
4.	CM10a, CM3
5.	CM10, CM10b, CM1
6.	CM10b, CM1b

GPI anchored proteins have been identified in *S. solfataricus *while in *M. barkeri *an archaeal ether-based phopholipid bearing the GPI anchor moiety head group has been identified [23,35]. Using the big-π-predictor program, Eisenhaber and co-workers also predict the likelihood of GPI anchor substrate proteins in a subgroup of archaean species including *A. pernix*, *A. fuldigus*, *M. thermoautotrophicum *and *P. horikoshii *[22]. However, no biochemical activity related to PIG-A has been demonstrated from archaeal sources until now. Our analysis suggests that either these organisms do not have any PIG-A protein *per se *or the proteins have diverged significantly as is the case with *G. lamblia*.

## Discussion

GPI biosynthesis plays a critical role in the biology of eukaryotic cells by providing membrane anchors to a large number of proteins and glycoconjugates involved in myriad functions, including signal transduction and pathogenesis [19]. PIG-A is an important gene in the biosynthetic pathway and studying its evolution may help us to understand how the GPI-biosynthetic pathway evolved in its present form in more complex organisms, and how it may be manipulated to obtain desired phenotypes.

From phylogenetic analysis, *G. lamblia *PIG-A appears evolutionarily closer to the archaeal PIG-A proteins than to those from other eukaryotes. However, there is one major difference. While archaeal PIG-A lacks any transmembrane domain, *G. lamblia *PIG-A has been predicted to possess a single transmembrane segment. This correlates with their intracellular localization. Archaeal PIG-A is a soluble, probably cytoplasmically localized protein, since archaeabacteria lack ER; while *G. lamblia *possesses rudimentary ER vesicles to which their PIG-A is targeted. Thus, the *G. lamblia *PIG-A acquired the transmembrane segment in response to increasingly complex cellular ultrastructure.

Phylogenetic analysis further demonstrated that PIG-A proteins of archaea and eukarya evolved from the glycosyltransferases and lipopolysaccharide glycosyltransferases of prokaryotes. Indeed, the results obtained using PLSR suggest that not only did PIG-A proteins evolve from glycosyltransferases but that the PIG-A protein from a primitive eukaryote like *G. lamblia *should more correctly be classified as glycosyltransferase. It also allowed us to verify that the motifs identified and analyzed by us could in fact be useful in making a distinction between 'true' PIG-A proteins and glycosyltransferases.

PIG-A proteins were found to have twelve conserved motifs, of which CM7 is highly promiscuous and cannot be used for identifying these proteins. ScanProsite analysis suggested that seven of the twelve motifs are present in glycosyltransferases, LPS and other glycosyl transferases. Of these, CM1, CM1a, CM1b, CM2c, CM3, CM4e, CM5e, CM10, CM10a, and CM10b were all found to be significant for identification of PIG-A proteins and their classification according to our PLSR analysis. However, CM1, CM1a, and CM1b were found to be the most robust of all variables in PLSR.

It may be pointed out that the motif (E/D) X_7 _(E/D) of CM10gt (as well as CM10 and CM10ar) is considered a characteristic of not only for PIG-A proteins and glycosyltransferases of GT4 family but also of α-mannosyltransferases [32]. Besides this, motifs, CM4gt, CM5gt, CM8gt, CM9gt, and CM11gt, are also present in all members belonging to the GT4 family of glycosyltransferases. Thus, it is evident that at least five motifs identified in PIG-A have their origins in glycosyltransferases belonging to GT4 family derived from bacteria and archaeabacteria. These motifs appear to have been modified, and additional conserved motifs such as CM6 appeared, as PIG-A evolved and diverged away from glycosyltransferases and LPS proteins. The PLSR method failed to identify any of these motifs as significant for classification of PIG-A proteins.

The CM6 motif, in particular, was found to be specific only for eukaryotic PIG-A proteins by the ScanProsite analysis. One possible explanation could be that some evolutionary changes took place during the formation of eukaryotic lineages and have been retained throughout evolution. These changes may be important for adaptation of the protein to the organellar structure and, therefore, explains its lack of usefulness as a marker for classification of PIG-A proteins as assessed by the PLSR method.

Unlike glycosyltransferases of the GT4 family, β-N-acetylglucosamine transferases of the GT28 family, show no similarity with PIG-A. Since GT4 and GT28 families, and perhaps all glycosyltransferases have evolved from a common ancestor involved in the cell wall synthesis of the primitive organism [36] further development of this primitive organism would have depended on the evolution of cell wall biogenesis enzymes. Therefore, a single glycosyltransferase would have probably evolved into many different classes of glycosyltransferases, each capable of a specific function.

The studies presented in this paper demonstrate that PIG-A proteins possess characteristic motifs that can be used for identifying PIG-A proteins from newly sequenced genomes. Further, these studies lay the foundation for site-directed mutagenesis and deletions experiments to understand the function of PIG-A proteins in the GPI anchor biosynthesis.

## Conclusion

Using a motif discovery approach and ScanProsite analysis, we identified eleven conserved motifs that are present in PIG-A proteins. A PLSR analysis suggests that the three motifs, [STC]-D-F-F-[YFC]-P-X-X-G-G-[VI]-E-X-H-X-[YF], D-[FTW]-[FHY]-[YFCP]-[PS]-X-X-[GD]-G-[VI] and [STC]-D-F-F-[YFC]-P-X-X-G-G-[VI] are the most robust for identification of PIG-A proteins. Statistical as well as phylogenetic analysis further demonstrates that PIG-A proteins evolved from glycosyltransferases. Additionally, our analysis suggests that PIG-A proteins from archaeabacteria and primitive eukaryotes like *G. lamblia*, that have been identified using BLAST, are in reality closer to bacterial GT4 glycosyltransferases than to eukaryotic PIG-A proteins and should be classified as such rather than as 'true' PIG-A proteins.

## Methods

### Sequence analysis of PIG-A

PIG-A sequence from *Homo sapiens, Entamoeba histolytica, Drosophila melanogaster, Dictyostelium discoidium, Saccharomyces cerevisiae, Schizosaccharomyces pombe, Candida albicans, Plasmodium falciparum*, and *Trypanosoma brucei *were obtained from the web site created by Eisenhaber *et al*. [37].

To identify the PIG-A homologus sequence in *Leishmania major*, we used the PIG-A sequence from *Homo sapiens *as the query. BLAST analysis was done against the *Leishmania major *gene database [38]. The sequence with the highest E value (3e-110) was selected and was cross-verified by BLAST analysis against the human genome.

Similarly, PIG-A from *Giardia lamblia, Arabdiopsis thaliana, Oryza sativa, Caenorhabditis elegans, Aeropyrum pernix *and *Thermoplasma acidiphilum *as well as other *Archael *genomes were identified.

Sequence analysis was done using Jalview version 2.2. We used MAFFT version 5 for ClustalW analysis. Gaps were removed after ClustalW alignment. These aligned sequences were then used for building phylogenetic trees. Rooted tree was built using TreeTop program, available from GeneBee Molecular Biology server [39], and unrooted tree was built using Phylodendron [40].

Motifs were identified manually as well as by using PRATT [41] and Gblocks software [28,29,42]. The motifs identified manually were represented in the Prosite format and were used to search the Swiss-Prot and TrEMBL databases with match mode of greedy, overlaps, and "no includes". The motifs were subsequently modified by deleting sequences from the ends and subjected once more to database search (Scan Prosite) in order to determine the specificity of these motifs in identifying PIG-A sequences.

### Statistical analysis using Partial Least Squares Regression

We assumed a linear model described in matrix notation as **y **= **Xβ + F **where, **X **is a matrix of independent variables, and **y **is some response variable to be predicted. **β **is the PLS regression coefficient vector and **F **the residuals estimated with a desired loss function. For PLSR the aim is to decompose **X **and **y **as:

(8) $$\begin{array}{l} X=SP'+E\\ y=UQ'+F \end{array}$$

where **S **(*N *× *K *matrix) and **U **(N × 1 matrix) are **X**- and **y**-score matrices respectively, **P **(*K *× *K *matrix) and **Q **(*K *× *K *matrix) are the corresponding loading matrices, **E **and **F **are residual matrices and **U **is related to **S **according to the inner relation:

(9) **U **= **SB + H**

where **B **is matrix containing the regression coefficients and **H **is a residual matrix.

Thus, **y **can be written as:

(10) **y **= SBQ' + F*

It should be noted that **y **in this case is a {-1/1} variable with -1 representing the 10 GT4 sample), and 1 representing the 43 PIG-A samples. Thus, zero is used as a threshold value where the predicted values of samples below zero are said to belong to the class -1 (GT4) and values above zero are said to belong to class 1 (PIG-A). Thus, using a {-1/1} labelling we can use PLSR as a method for discrimination analysis (DA) and PLSR used in this mode is often labelled as PLS-DA.

For methods like PLSR, one of the important aspects is to find the optimal number of PLS components (A_Opt_), preferably from a suitable validation method like cross validation (CV) or independent test set validation.

When CV is applied in regression, A_Opt _is determined based on prediction of kept-out samples from the individual models. The root mean square error (RMSE) is an error measure for how well the model performs, and is given by the expression

(1) $$RMSE=\sqrt{\frac{\sum_{n=1}^{N}{\left(y-\hat{y}\right)}^{2}}{N}}$$

When representing estimation of future prediction error this is called RMSEP. The notation RMSEP_CV _is used to indicate the error of prediction estimated by cross validation. RMSEC is the fit from the calibration. Normally, one would chose A_Opt _from the lowest RMSEP value, but this can lead to overfitting and an unnecessary high number of components.

### Uncertainty estimates in β and variable selection

The approximate uncertainty variance of the PCR and PLS regression coefficients *b *can be estimated by jack-knifing.

(2) s2b=(∑m=1M(β−βm)2)((N−1)N)

where

*N *= the number of samples

*s*^2^*β *= estimated uncertainty variance of individual regression coefficients, *b*

*β *= the regression coefficient at the cross validated A_Opt _components using all the *N *samples

*β*_*m *_= the regression coefficient at the rank A using all objects except the object(s) left out in cross validation segment *m*.

The degrees of freedom used here is *N*. Another alternative is to use the number of segments, *M*, when CV alternatives other than full CV are applied. In this case *M *may also replace *N *in equation (2).

On the basis of such jack-knife estimates of the uncertainty of the model parameters, useless or unreliable variables may be eliminated automatically, in order to simplify the final model and making it more reliable. This is done by significance tests, where a t-test is performed for each element in ***β ***relative to the square root of its estimated uncertainty variance *s*^2^*β*, giving the significance level for each parameter. This approach has been termed as "JK-PLSR"[43].

### Validation of the calibration model and the selected genes

The importance of proper validation is appropriately addressed by Ambroise and McLachlan (2002) [44] as well as Wood, Visscher, and Mengersen (2007) [45], clearly showing the effects of selection bias during modelling and subsequent prediction. As per the recommendations made in the two articles sited above, we have used external or two-level cross-validation for determining the real predictive value of the selected motifs. We have termed our procedure for external validation as double CV (DCV), basically adding an extra or outer layer of validation on top of the normal CV procedure, hence the name DCV.

We begin by randomly selecting *q *samples for each DCV segment (for e.g. diving the data set in M non-overlapping subsets of roughly equal size) taking care to include the same number of samples (along with any replicates) from each class in the DCV segments as in the original population. Begin with subset M_*i*_, *i *= 1...*u*, M_i _constitutes the outer layer containing *q *samples; typically *u *= 10 representing 10-fold DCV. The remainder samples (representing the inner layer), N_*j *_= N-*q*, are then used for building the calibration model and variable selection, for example using the regular *k*-fold CV. The motifs thus selected (from the inner layer) are subsequently used to predict the *q *samples in DCV segment, or the outer layer M_i_.

The DCV procedure is repeated until all samples have been included at-least once in the outer-layer. It should be noted, that in contrast to the standard *k-*fold CV giving a single model and a single set of selected variables, our procedure generates a total of *i *(here *i *= 10) sub-models giving prediction errors for both the inner and outer layer, as well as *i *= 10 set of important variables from each of the DCV segments. Results from the analysis can be presented at two different levels: (1) average errors for all the inner layers, the overall calibration error, reported with and without variable selection and (2) average errors for the outer layers (for the selected variables only), the overall prediction error.

Comparing the results obtained at the two levels of validation outlined above, it should be noted that the overall prediction error of 23% based on DCV is comparable to the aggregate of prediction error of 21.6% for the inner layer CV without any variable selection. However, the inner layer CV with variable selection give a prediction error of only 17%. Thus, showing the dangers of reporting downwards-biased error rates if proper validation routines are not followed [45].

Finally, the important variables were extracted as a set of motifs appearing in maximum number of the DCV segments varying from variables appearing in all the *i *= 10 DCV segments to at-least one single DCV segment. As a rule of thumb, variables common in at least 50% of the DCV segments are reported.

## Authors' contributions

**NO **identified the PIG-A sequences from eukaryotes, and archaea. She also identified the glycosyl transferase sequences from prokaryotes and archaea. **NSS **did the statistical analysis using partial least square regression. **AB **helped in drafting the manuscript and critically evaluated it for the intellectual content. **RM **and** SSK **provided the concept for the paper, participated in sequence alignment, identification and validation of the motifs, generation of the matrix for statistical analysis, and drafting the manuscript. All authors read and approved the final manuscript.

## Supplementary Material

Additional file 1 **Phylogenetic analysis of PIG-A proteins from eukaryotes using bootstrap values**. The bootstrap values in most cases were well over 50 and are hence may be treated as reliable estimates of the evolutionary relationship between PIG-A of different eukaryotes. Click here for file

Additional file 2 **Phylogenetic analysis of PIG-A proteins from eukaryotes and archaeabacteria using bootstrap values**. The bootstrap values in most cases were well over 50 and are hence may be treated as reliable estimates of the evolutionary relationship between PIG-A of eukaryotes and archaea bacteria. Click here for file

Additional file 3 Conserved motifs in PIG-A sequences from eukaryotes identified using Gblocks software. Click here for file

Additional file 4 Conserved motifs in PIG-A sequences from eukaryotes and archaea identified using Gblocks software. Click here for file

Additional file 5 **Phylogenetic analysis of PIG-A proteins and glycosyltransferases using bootstrap values**. The bootstrap values in most cases were well over 50 and are hence may be treated as reliable estimates of the evolutionary relationship between PIG-A and glycosyl transferases of different organisms. Click here for file

Additional file 6 Matrix used for PLSR analysis. Click here for file
