# Supplementary figures and images for: Unique motifs identify PIG-A proteins from glycosyltransferases of the GT4 family
Source: BMC Evol Biol. 2008 Jun 4;8:168. doi: 10.1186/1471-2148-8-168 (PMC2446393; doi:10.1186/1471-2148-8-168)

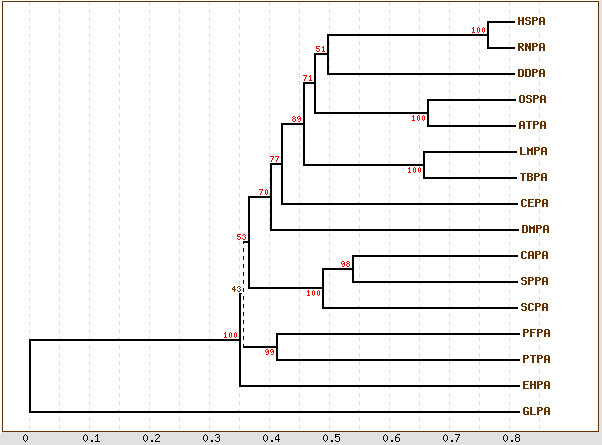

Supplement: Additional file 1 — Phylogenetic analysis of PIG-A proteins from eukaryotes using bootstrap values. The bootstrap values in most cases were well over 50 and are hence may be treated as reliable estimates of the evolutionary relationship between PIG-A of different eukaryotes. [file 1471-2148-8-168-S1.doc]

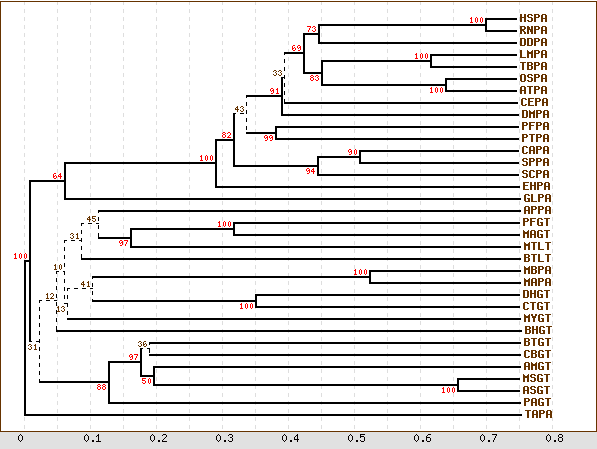

Supplement: Additional file 5 — Phylogenetic analysis of PIG-A proteins and glycosyltransferases using bootstrap values. The bootstrap values in most cases were well over 50 and are hence may be treated as reliable estimates of the evolutionary relationship between PIG-A and glycosyl transferases of different organisms. [file 1471-2148-8-168-S5.doc]
