# Supplementary material for: Unique motifs identify PIG-A proteins from glycosyltransferases of the GT4 family
Source: BMC Evol Biol. 2008 Jun 4;8:168. doi: 10.1186/1471-2148-8-168 (PMC2446393; doi:10.1186/1471-2148-8-168)
Supplement: Additional file 3 — Conserved motifs in PIG-A sequences from eukaryotes identified using Gblocks software. [file 1471-2148-8-168-S3.doc]

Additional 3. Conserved motifs in PIG-A sequences from eukaryotes identified using Gblocks software.

| **BLOCK** | **SEQUENCE** |
| --- | --- |
| 1 | [VILA]-[STC]-D-F-F-[YFC]-P-[NQGCS]-X-G-G-[VI]-E-X-H-[IVLQ]-[YF]-X-[LI]-[SAG]-[QLHK]-[CKRMFN]-[LF]-[ILMV]-X-X-G-[HFNL]-[KRS]-[VI]-[IV]-[ITV]-[VIML]-T-[HRN]-[AQNFSGK]-[YN]-[GSKQN]-X-[RTC]-X-G-[VI]-R-[YHVW]-[LVMY]-[TSKG]-[SNG]- [GY]-[LIM]-[KT]-V-Y-[YH]-[LVIC]-P |
| 2 | [PLA]-X-X-[RS]-X-[ILV]-[FLVH]-X-[RE]-[EH]-X-[IVF]-X-[IV]-[ILV]-H-[SGAC]-H-X-X-[FLATY]-S-X-[MLFI]-X-[HGL]-[DEQT]-[AGSTLF]-[LIM]-X-[HIVFW]-[AG]-X-[TLSA]-[[MLV]-G-[LYIF]-[KPQRS]-[TAV]-[VFCIL]-[FLY]-T-[DE]-H-S-[LM]-[FYA]-[GASR]-[FLG]-[ANKSD]-[DENGV]-X-X-[SACE]-[VIFAM]-X-X-[NS]-[KP] |
| 3 | [LCR]-X-X-X-[LFMI]-X-X-X-[DNGSH]-X-X-I-[CAS]-V-S-X-[CEIV]-[STCGN]-[KRE]-[ED]-N-[TML] |
| 4 | [VISCL]-[LIVM]-R-[ATSGH]-X-X-X-[PQK]-X-X-[VIA]-[SFYD]-[VIMT]-[VI]-[PG]-N-[AI]-[VLTI] |
| 5 | [IV]-[VAI]-[VIF]-[VILMA]-[STGQ]-R-[LM]-[VYFT]-[YPQF]-[RN]-K-G-X-D-L-[LIFA]-X-X-[IVL]-[PQG] |
| 6 | [FWV]-[ILVY]-[VI]-[GAV]-G-[EDNS]-G-P-[KMR]-[RFSHKM]-X-X-[LFV]-[EQK]-[VMTIL] |
| 7 | [EP]-X-X-X-[LMK]-X-X-[RQS]-[VTL]-X-X-[LV]-G-X-X-X-[HGQN]-X-X-[VT]-[RK]-X-[VFIL]-[LM]-X-X-[GC]-[HDQ]-I-[FYG]-[LIV]-[NHI]-X-S-[LY]-[TL]-E-[AG]-[FY]-[CGS]-X-[AVIS]-[IL]-[VIL]-E-[AS]-[AL]-[SQ]-[CE]-[GNA]-[LC]-X-[VICP]-[VI]-[STA]-[TS]-X-V-G-G-X-[PDSK]-[ES]-V-[LY]-[PK] |
| 8 | Y-X-[WP]-X-X-[VI]-[AS]-X-[RK]-[TV] |

Highlighted sequences represent:

Yellow underlined: CM1

Blue underlined: CM2

Green underlined: CM3

Yellow: CM4

Green: CM5

Blue: CM6

Pink: CM7

Olive: CM8

Light grey: CM9

Red: CM10

Dark grey: CM11

Dark green: CM 12

Block 1 was obtained when PIG-A sequence from *G. lamblia* was not used in the analysis. Gblock software was configured such that it allowed smaller blocks.
