# Supplementary material for: Unique motifs identify PIG-A proteins from glycosyltransferases of the GT4 family
Source: BMC Evol Biol. 2008 Jun 4;8:168. doi: 10.1186/1471-2148-8-168 (PMC2446393; doi:10.1186/1471-2148-8-168)
Supplement: Additional file 4 — Conserved motifs in PIG-A sequences from eukaryotes and archaea identified using Gblocks software. [file 1471-2148-8-168-S4.doc]

Additional file 4. Conserved motifs in PIG-A sequences from eukaryotes and archaea identified using Gblocks software.

| BLOCKS | SEQUENCES |
| --- | --- |
| 1 | D-[FWT]-[FHY]-[YFCP]-[PS]-X-X-[GD]-[G]-[VI]-[EQS]-X-[HSY]-[ILVQM]-[YFRHK]-X-[HSY]-[ILVQM]-[YFRHK]-X-[LIV]-[SGATK]-[QLHKR]-X-[LF]-X-X-X-G-[HNLFY]-[KSDER]-[VI]-X-[ITV]-[VIMTF]-[TS]-[HRNV]-[ANSGKQFCT]-[YNLG]-[GKSQNPD] |
| 2 | [RTCK]-X-[GDH]-[VIN]-[RKSAV]-[YVHWDI] |
| 3 | [PASKRL]-X-X-[RFYS]-X-[ILEA]-[FVLIH] |
| 4 | [RSDE]-[ELFGH]-X-[IVPYF]-X-[IV]-[IV]-[HN]-X-H |
| 5 | [MLFIT]-X-[HGLMS]-[DEQLIVT]-[AGSTLF]-[LIMY]-X-[HIFAVW]-[AGS]-[KRGS]-[TLSLDKRA]-[MLIV]-G-X-X-[TVRSPA]-[VCFI]-[FLYAV]-T-[DENF]-H-[ST]-[LIDM]-[FYVA]-[ANKSYTMD]-[DEGPKV]-X-X-[SACLIE]-[VIFALYNM] |
| 6 | [LCTGR]-X-X-X-[LIVFM] |
| 7 | [NGDSQRH]-X[VIAST]-[IM]-[CSA]-S |
| 8 | [TVICAE]-X-[KRAE]-[EDK]-[NKDY]-[TMLS] |
| 9 | [VITR]-[SFYVD]-[VMTIK]-[IL]-[PFG]-[NL]-[AGFI]-[VLTIM] |
| 10 | [DIVES]-X-X-X-[FKYS]-X-[PIYH] |
| 11 | [IV]-[VILA]-[VFITY]-X-X-R-[LMI]-[VFYT]-[YPFWRKVQ]-[RNKD]-K-G-X-[DHQY]-[LVNR]-[LIFAV]-X-X-X-[IVLFA]-[PQREG] |
| 12 | [PQEKRM]-X-X-X-[FWLV]-X-[IVM]-[GAIV]-G-[EDNKS]-G-[PE]-[KRMFLE]-X-X-X-[LFIV]-[EQKR]-X-[VMITL] |
| 13 | [EQKAP]-X-X-X-[LMK] |
| 14 | [RQSN]-[VTFL]-X-X-[LVT]-G-X-[LIV]-X-[HGQERDN]-X-X-[VTK]-[RKPLM]-X-[VFILY]-[LMY]-X-X-[GAC]-X-[IVL]-[FYTG]-[LAV]-[NHVLYI]-X-S-[LISY] |
| 15 | [TNDAL]-[ED]-[AGST]-[FY]-[CGS]-[MTI]-[ILAV]-X-E-[AS]-[ALMI]-[SAQ]-[CSKE]-[GNEA]-[LTVC]-X-[VITCP]-[VIM]-[STAV]-[TSM]-X-[VQNHD]-[GSF]-[GP]-X-[PSKD]-[EDS]-[VIN] |
| 16 | [LMIFA]-X-X-X-X-X-X-[AVTLI]-[IVLQ] |
| 17 | Y-X-[WLP]-X-X-[VIH]-[AVCYIS]-X-[RSKQ]-[TILV] |

Same colour scheme has been used as for Additiona file1. Gblock software was configured to allow for smaller blocks.
