# Supplementary material for: Unique motifs identify PIG-A proteins from glycosyltransferases of the GT4 family
Source: BMC Evol Biol. 2008 Jun 4;8:168. doi: 10.1186/1471-2148-8-168 (PMC2446393; doi:10.1186/1471-2148-8-168)
Supplement: Supplementary file 5 [file 1471-2148-8-168-S2.doc]

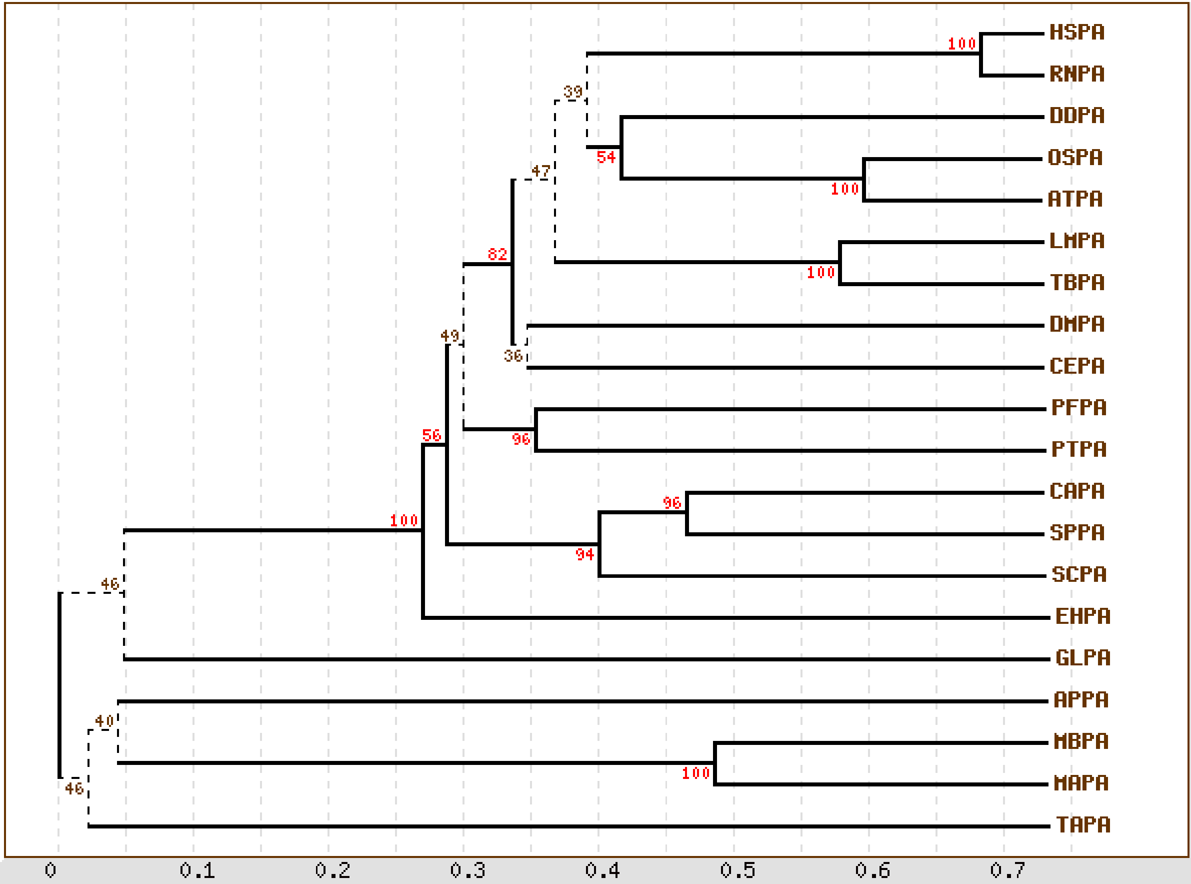
HSPA
RNPA
DDPA
OSPA
ATPA
LMPA
TBPA
DMPA
CEPA
PFPA
PTPA
CAPA
SPPA
SCPA
EHPA
GLPA
APPA
MBPA
MAPA
TAPA
100
39
54
100
47
100
82
49
36
96
56
96
94
100
46
40
100
46
0
0.1
0.2
0.3
0.4
0.5
0.6
0.7
